# A pilot trial of Convolution Neural Network for automatic retention-monitoring of capsule endoscopes in the stomach and duodenal bulb

**DOI:** 10.1038/s41598-020-60969-5

**Published:** 2020-03-05

**Authors:** Tao Gan, Shuaicheng Liu, Jinlin Yang, Bing Zeng, Li Yang

**Affiliations:** 1 Department of Gastroenterology and Hepatology, West China Hospital, Sichuan University, Chengdu, 610041 Sichuan China; 2 School of Information and Communication Engineering, University of Electronic Science and Technology of China, Chengdu, 611731 Sichuan China

**Keywords:** Image processing, Colonoscopy, Oesophagogastroscopy, Computer science

## Abstract

The retention of a capsule endoscope (CE) in the stomach and the duodenal bulb during the examination is a troublesome problem, which can make the medical staff spend several hours observing whether the CE enters the descending segment of the duodenum (DSD). This paper investigated and evaluated the Convolution Neural Network (CNN) for automatic retention-monitoring of the CE in the stomach or the duodenal bulb. A trained CNN system based on 180,000 CE images of the DSD, stomach, and duodenal bulb was used to assess its recognition of the accuracy by calculating the area under the receiver operating characteristic curve (ROC-AUC), sensitivity and specificity. The AUC for distinguishing the DSD was 0.984. The sensitivity, specificity, positive predictive value, and negative predictive value of the CNN were 97.8%, 96.0%, 96.1% and 97.8%, respectively, at a cut-off value of 0.42 for the probability score. The deviated rate of the time into the DSD marked by the CNN at less than ±8 min was 95.7% (*P* < *0.01*). These results indicate that the CNN for automatic retention-monitoring of the CE in the stomach or the duodenal bulb can be used as an efficient auxiliary measure in the clinical practice.

## Introduction

Capsule endoscope (CE) has become one of the best diagnostic tools for diagnosing small intestinal diseases because of its painless and non-invasive nature^1–4^, but it has some weaknesses. One of these is that after the CE is swallowed, its movement in the digestive tract is completely dependent on gastrointestinal motility, especially the gastroduodenal emptying velocity. If the gastroduodenal emptying velocity is too slow, the CE can become stagnated in the stomach or duodenal bulb for several hours, which can cause energy loss of the built-in battery. Thus, examination of the whole small intestine may not be finished. How to predict the residence time of the CE in the stomach or duodenal bulb has not been solved, and medical staff may have to wait for several hours in the examination room to monitor whether the CE enters the descending segment of the duodenum (DSD)^5,6^. If the CE cannot enter the DSD in 2–3 h, some interventions, e.g., drugs or gastroscopy, can be used to push the CE forward into the DSD^7^, which is a tedious and boring task, especially for some patients who have to undergo the CE examination at the same time, which could greatly increase the monitoring workload for the medical staff.

Artificial intelligence (AI), as a new technique, has been developed in the recent years, which includes Autoencoder^8^, Deep Belief Network^9^, Convolution Neural Network (CNN)^10^, and Deep Residual Network^11^, and they have been used in the medical image analysis and have been proved to be effective in some medical diagnostic fields, such as pulmonary nodules^12^, breast lesions^13,14^, skin cancer^15^, early gastrointestinal cancers^16,17^, polyps^18^, and small-bowel diseases^19–23^.

Of those techniques, the CNN^24^ is a type of deep learning mode^25–27^ that requires the preprocessing of the image data inputted as a training image set for extracting specific features and quantities by using the multiple network layers (convolutional layers, fully-connected layers), and then iteratively changed through the multiple convolutions and the non-linear operations until the training data set is converted into a probability distribution of the potential image categories. With its high efficiency in the image analysis, the CNN has become a principal method of deep learning for images. In this study, we aimed to develop and validate the CNN-based program for the automatic recognition of the DSD as the end of the gastric retention during the CE examination.

## Outcome Measures

The primary outcome was the ability of the CNN to distinguish the DSD from the stomach/duodenal bulb using an area under the receiver operating characteristic (ROC) curve for the probability score, sensitivity, specificity, positive predictive value, and negative predictive value of the CNN recognition capability for the DSD and the stomach/duodenal bulb. The secondary outcome focused on the clinical evaluation of the point of deviation time into the DSD marked by the CNN compared with that confirmed by the endoscopists.

First, we manually annotated all the images of the DSD and the stomach/duodenal bulb. The CNN was trained to distinguish the DSD from the stomach and stomach/duodenal bulb in the validation set and outputted the probability score (range, 0–1). The higher the probability score, the more confidence the CNN had in its recognition of the DSD, but on the contrary for the stomach/duodenal bulb.

We evaluated the algorithm as follows: 1) If the CNN had a probability score larger than the cut-off value on an actual image of the DSD, it could be considered a true positive, and an image of the non-DSD with a probability score larger than the cut-off value could be considered a false positive; 2) If the CNN had a probability score less than the cut-off value on an actual image of the stomach/duodenal bulb, it could be considered a true negative, and an image of the non-stomach/duodenal bulb with a probability score less than the cut-off value was considered a false negative.

### Second, the clinical evaluation of the point of deviation time is listed below

After the CE examination, a disc of the images was sent back to our endoscopic center for a further analysis, and the standard images of the CE into the DSD were scanned and confirmed by two CE endoscopists. If the results were inconsistent, a third CE endoscopist was consulted. After the image was confirmed, the time point was regarded as the entry time of the CE into the DSD. The time point of the first image of the CE into the DSD marked by the CNN was determined by the computer software and was compared with that confirmed by the CE endoscopists (standard time). The deviation time was limited to ±1 min, ±3 min, and ±8 min. If the deviation time range was greater than ±8 min, it was considered a failure.

### Statistical analysis

Statistical analysis was performed with the medical statistical software. The correlation between the two variables was evaluated using Pearson’s *χ*^2^, Fisher’s exact test, and *t-* test. *P* < 0.05 was considered statistically significant. The ROC curve was plotted by varying the threshold of the probability score, and the area under the curve (AUC) was calculated for assessing the distinction. The sensitivity, specificity, positive predictive value, and negative predictive values of the CNN’s ability to distinguish the DSD from the stomach/duodenal bulb were calculated using the cut-off values for the probability score according to the Youden index. The value of Youden index was the sum of the sensitivity and the specificity minus 1, and the cut-off value was the probability score corresponding to the maximal value of the Youden index.

## Results

Data from a total of 115 patients undergoing the CE into the DSD (57 outpatients, 58 inpatients; 62 male, 53 female; 54.9 ± 15.7 years, range 18–84 years) were collected for clinical validation in this study. All of them had undergone gastroscopy and colonoscopy at least once before the CE examination, but no obstruction or significant lesions were found. Therefore, the CE examination was performed at our endoscopic center to discern whether the lesions existed in the small bowels. The categories of small-bowel diseases in the patients were enteritis, small intestinal tumor, polyp, bleeding, Crohn’s disease, parasite disease, angioectasia, etc.

### The recognition results of the CNN for the DSD and stomach/duodenal bulb

**Figure 1:**
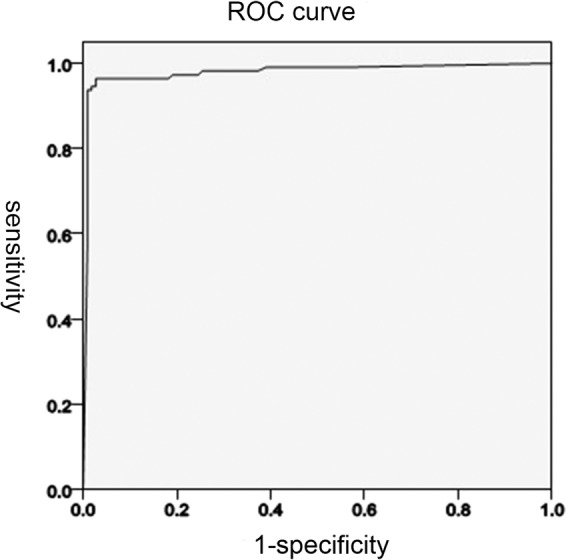
The receiver operating characteristic curve (ROC) of this study.

The average operational speed of the AI software was 0.426 millisecond per image (ms/image) in the 115 patients, which was satisfactory for auto-monitoring. The ROC curves for the probability score are shown in Fig. 1. The AUC of the CNN used to distinguish the DSD was 0.984. The cut-off value for the probability score was 0.42.

At this cut-off value, there were 9730 true positives and 270 false negatives among the 10,000 DSD images, and 360 false positives and 9640 true negatives among the 10,000 stomach/duodenal bulb images. Therefore, the sensitivity, specificity, positive predictive value, and negative predictive value of the CNN were 97.8%, 96.0%, 96.1%, and 97.8%, respectively (Table 1).

**Table 1:** Classification of image by CNN technology.

CNN classification	Endoscopists’ classification		Total
	DSD	Stomach/duodenal bulb	
DSD	9780	400	10180
Stomach/duodenal bulb	220	9600	9820
Total	10000	10000	20000

Sensitivity 97.8%, Specificity 96.0%.

**Figure 2:**
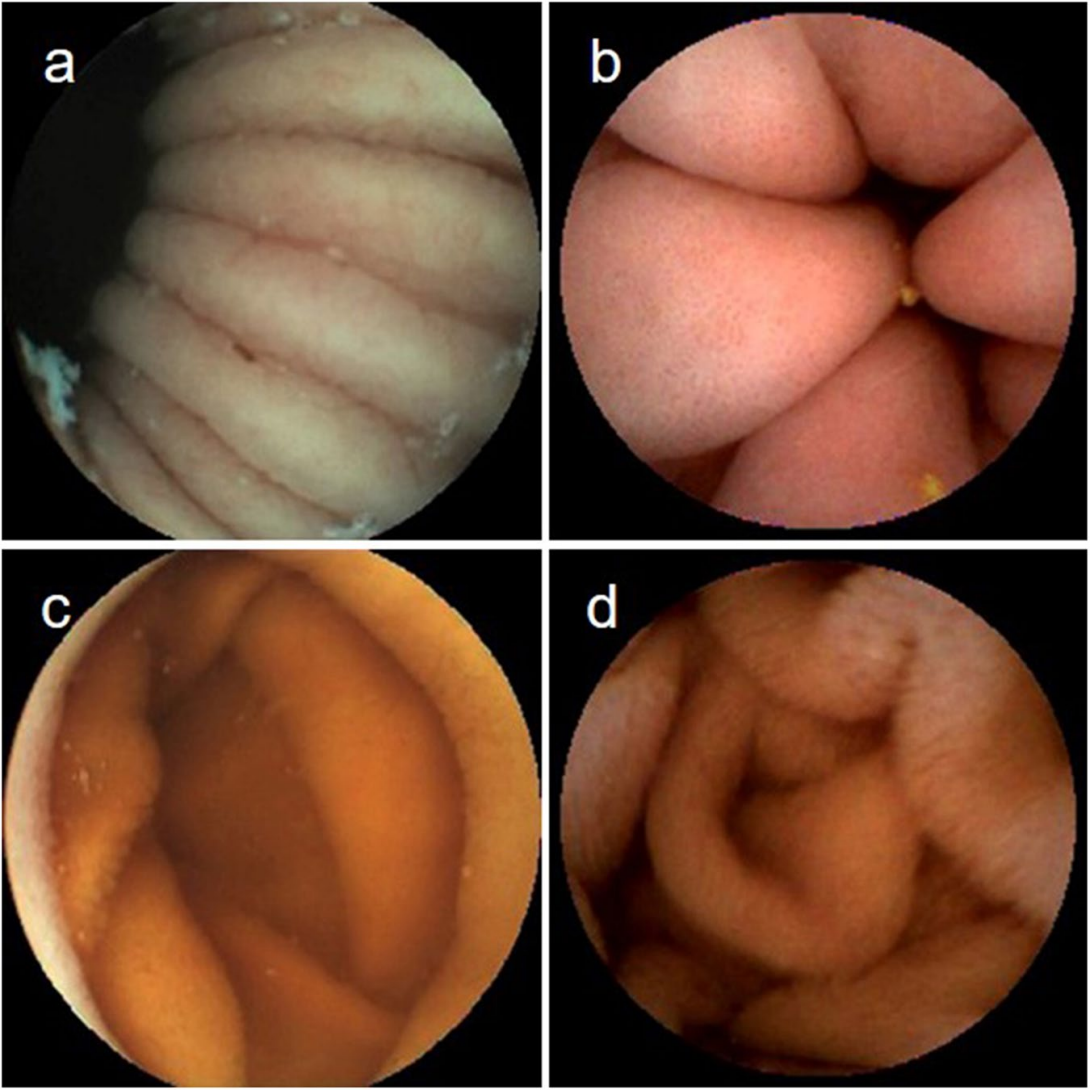
The images of the stomach and duodenum. (**a**) Image of the gastric body mucosa. (**b**) Image of the gastric antrum mucosa. (**c**) Image of the duodenal bulb mucosa. (**d**) Image of the DSD mucosa.

Representative stomach/duodenal bulb images and the DSD images were correctly recognized by the CNN (Fig. 2a–d).

False negative images (n = 270) were classified into five categories according to the interference factors of the false negative situation (Table 2), and the false positive images (n = 360) were classified into six categories according to the interference factors of the false positive situation (Table 2).

**Table 2:** Interference factors of classification discrepancies by the endoscopists and by CNN.

False-negative image	270 (100%)
Poor focus	2 (0.7)
Laterality/partialness*	65 (24.1)
Foam	3 (1.1)
Bile fluid	6 (2.2)
Fold	194 (71.9)
False-positive images	360 (100%)
Poor focus	3 (0.8)
Laterality/partialness*	77 (21.4)
Foam	5 (1.4)
Bile fluid	15 (4.2)
Fold	257 (71.4)
Debris	3 (0.8)

*Image of partial or lateral wall mucosa of stomach/duodenum/DSD.

Data represented as number (%).

**Figure 3:**
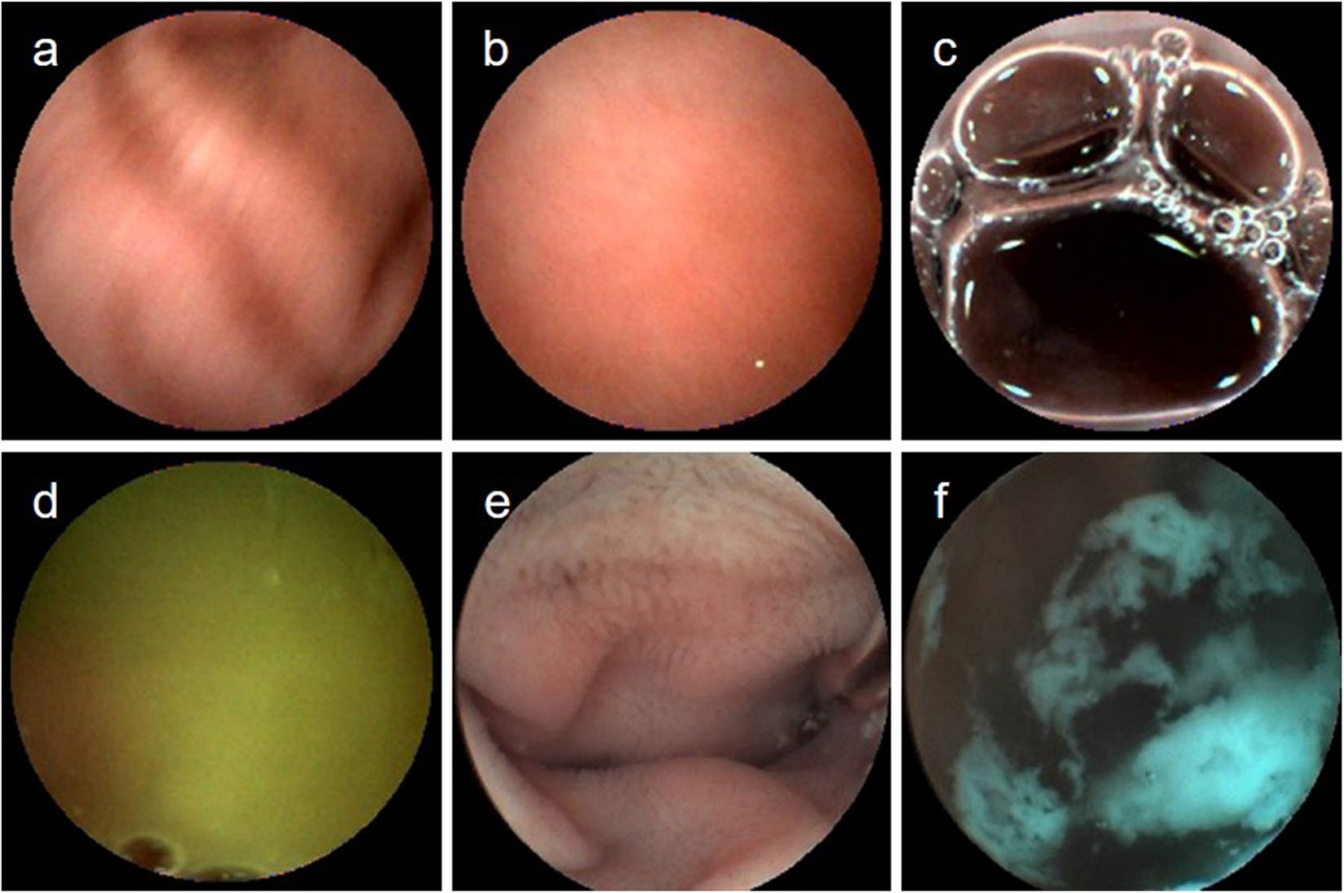
The interference factors. (**a**) Image of the obscure view (Poor focus). (**b**) Image of the gastric wall mucosa (laterality, partialness). (**c**) Image of the foam. (**d**) Image of the bile fluid. (**e**) Image of the misjudged fold. (**f**) Image of the debris.

The interference factors included poor focus, laterality/partialness, foam, bile fluid, fold, and debris (Fig. 3a–f), the majority of which were laterality/partialness and fold.

### The results of deviation time marked by the CNN

The monitoring results showed that the deviation time into the DSD was 1.41 ± 1.17 min, range 0.1–7.0 min in 110 patients (deviation time less than 8 min). In the 115 patients, the deviation rates of the time marked by the CNN of < ± 1 min, ±1–3 min, and ≥ ±3 min were 39.1% (45 patients), 50.4% (58 patients), and 10.4% (12 patients), respectively (*P* < *0.01*) (Table 3).

**Table 3:** The deviation time in different patients.

Time point of deviation	< ± 1 min,	± 1 − ± 3 min	> = ± 3 min
Time of advance	45	58	12
Time of delay	70	57	103
*P*		**<**0.01	

Of the 115 patients, 5 had the deviation time >8 min (1 male, 4 female), 4 had the deviation time of 12.8–55.5 min ahead of the standard time, and 1 had the deviation time of 24.2 min behind the standard time. The duration of the interference was >10–35 min and the duration of the stagnation was >5–10 min.

The monitoring result showed that when the interference and the stagnation occurred between the time marked by the CNN and the standard time, the deviation time was positively correlated with the duration of the interference and the stagnation (*P* < *0.01*) (Table 4).

**Table 4:** The influence factors of deviation time.

DT* (sec)	Time of interference (sec)			Time of stagnation (sec)		
	<20	20–50	>50	<20	20–50	>50
<60	55	10	1	62	4	0
60–120	14	18	5	16	21	0
120–180	2	3	2	3	3	1
*P*	<0.01			<0.01		

*DT: deviation time marked by CNN.

## Discussion

The CE plays an important role in diagnosing such small-bowel disease as obscure bleeding^28–30^, Crohn’s disease^31^, and tumors, but the gastroduodenal retention remains to be a problem in some patients. If the CE passes through the stomach and duodenal bulb into the DSD too slowly, the medical staff have to be continuously monitoring even for hours, and they have to discern whether the long retention of the CE occurred in the stomach for fear of the battery exhausted.

Now a computational method has been developed for detection of a broader spectrum of the lesions in the small bowels^19–23^, such as obscure gastrointestinal bleeding, erosions, ulcers, and angioectasia. From the results of this research, the sensitivity and specificity was >85% on the whole. This new diagnostic computational method is based on the CNN, which can automatically detect the lesions in the CE images so as to greatly relieve the overload of the medical staff’s monitoring. We have also reported the automatic detection of hookworm^22^ and other diseases^32^ in the small bowels by the computational image procession with a sensitivity and specificity >70%. Without automatically monitoring the CE retention in the stomach/duodenal bulb and judging the CE into the DSD, the automatic detection of the small-bowel lesions cannot be performed. Therefore, in this study, we developed and validated the CNN-based method for automatic retention-monitoring of the CE in the stomach/duodenal bulb. We used 180,000 CE images for training and 20,000 independent wireless CE (WCE) images for testing. Our study revealed that the proposed CNN-based method achieved an excellent recognition effect for the DSD and stomach/duodenal bulb in the CE still frames. Our results showed that the CNN-based method had adequate sensitivity and specificity for clinical validation. Different from the other diagnostic trial methods of AI, the recognition results in this trial were correlated with the end of the retention monitoring and the patient departure from the examination room, and therefore, we focused on the actual clinical validation. The general results of this pilot trial are promising. Compared with the time points of the CE entering the DSD as confirmed by the endoscopy experts, whether the CE had reached the DSD was effectively determined in over 95% of the patients, with the deviation time of less than 8 min. With the development of AI technology in recent years, the CNN’s image processing speed (0.426 ms/sheet) is much greater than the filming speed (500 ms/sheet) of the CE. The recognition of the capsule image by the CNN would not be paused or delayed. The CNN makes it possible for us to automatically monitor the movement of the capsule in the stomach. If the time limit of the DSD recognition is given, e.g., the image of the DSD not recognized within 2 hours, a warning signal will be sent out by the software system to remind the gastric retention of the CE. If the effect is good, it can help the medical staff avoid the time-consuming and boring work.

In this study, the high efficiency of the CNN image recognition resulted not only from the improvement of the CNN technology itself, but also from the characteristics of this study. Large numbers of samples could be easily collected for the training to build a better application model and the recognition error could be corrected partly through the CNN algorithm. As a rule, the CE will not return to the duodenal bulb or the stomach when it has entered the DSD. Therefore, where the time of the CE entering the DSD was marked by the CNN, the images behind the marked time should be the DSD images. However, if later images were again judged to be images of the stomach or duodenal bulb, especially for some images which were confirmed repeatedly, the marked time point could be wrong and should be corrected again.

Though the result of the CNN in recognition of the CE entering the DSD was good enough in most of the patients, there existed a few patients who had a recognition failure. Analysis was performed on the images between the time point marked by the CNN and the standard ones, which showed that the deviation time was positively correlated with the duration of interference factors (Table 2) (*P* < 0.01). The longer the interference time was, the longer the deviation time was. The reason for the deviation caused by interference was that the CNN also had some difficulty categorizing some of the interference images, such as images of fold and laterality/partialness. If interference images of laterality/partialness continuously existed with no images of the stomach or duodenal bulb mucosa confirmed, or only blurry images of fold could be found, the interference images might be categorized as those of the DSD, which would result in a time point ahead of the standard one, or even a complete failure to judge the time point of the DSD. This would be a key point for algorithm improvement in the subsequent researches.

Analysis also showed that the deviation time was positively correlated with the duration of the CE stagnation between the time point marked by the CNN and the standard ones (*P* < 0.01). The longer the stagnation was, the greater the deviation time was. Deviation time due to stagnation had two points: If stagnation occurred in the duodenal bulb and the image of the DSD appeared late, the fold of the duodenal bulb, similar to the DSD, could be misjudged to be the DSD by the CNN, especially where the villi existed. Also the time point of the first stagnation image might be judged as the time point of the CE into the DSD, which could lead to the advance of the time point of the CNN. However, if the stagnation occurred in the DSD, without clear fold of the DSD confirmed or only a laterality/partialness of the DSD found, the time point of the last stagnation image might be judged as the time point of the CE into the DSD, which could lead to the delay of the time point from the CNN. This is another key point for algorithm improvement in the subsequent researches.

Our study still has some limitations. First, this was a retrospective study conducted at only one medical unit. This retention-monitoring system should be validated in other hospitals. Second, although numerous sample images were collected for training, more training images might achieve a more accurate diagnostic ability for the CNN. Second, only 115 patients were contained for clinical validation in the first step, and the further testing material was not prepared for the evaluation process due to a lack of patients in this study, which could cause an overestimation of the model performance and a lack of general appraisement of this developed AI. Third, our CNN system was investigated only using the OMOM system, and it is uncertain whether this CNN can be applied effectively to other CE systems. Fourth, the recognition of interference images and stagnation images was limited. Despite these limitations, the potential usefulness of the CNN system has been proved to be encouraging in distinguishing the stomach/duodenal bulb from the DSD in the CE images.

In conclusion, this pilot study has indicated that the CNN for automatic retention-monitoring of the CE in the stomach or duodenal bulb can be used as an efficient auxiliary measure for clinical practice. By combining this high-speed, high-accuracy system with the AI diagnostic system of the CE, we have a desire to develop a whole software that can be used not only for automatically monitoring the retention of the CE but also for automatically analyzing the CE images concerned.

## Materials and Methods

We retrospectively included 443 patients (the training image set: 328 patients, the validation image set: 115 patients) who had undergone the CE examination on suspicion of suffering from small-bowel diseases at the West China Hospital, Sichuan University. Data for each patient were obtained from a retrospective medical record review stored in the computer database. The examination was performed using an OMOM CE device (Jinshan, Chongqing, China). This clinical trial was reviewed and approved by the Ethics Committee of West China Hospital, Sichuan University, and the register number was 2018(92). The methods in this clinical trial were carried out in accordance with the guidelines and regulations of Chinese Clinical Trial Registry, and the register number was ChiCTR1800017785. All the patients signed the informed consent. None of them refused or withdrew from the examinations during the research period.

We developed a deep learning-based AI model for the endoscopic mucosa images of the DSD. For the algorithm to distinguish the DSD, the still images of the DSD captured by the CE were retrospectively collected, which were totaled 180,000 images (DSD 90,000, stomach/duodenal bulb 90,000) from the 328 patients as the training data set. We trained the model on the images of the DSD when captured by the OMOM CE device.

To evaluate the recognition accuracy of the constructed CNN, an independent test data set of the DSD images were collected totaling 20,000 images from the 115 patients as a validation image set. Of them, 10,000 images originated from the DSD and 10,000 images originated from the stomach and duodenal bulb.

**Figure 4:**
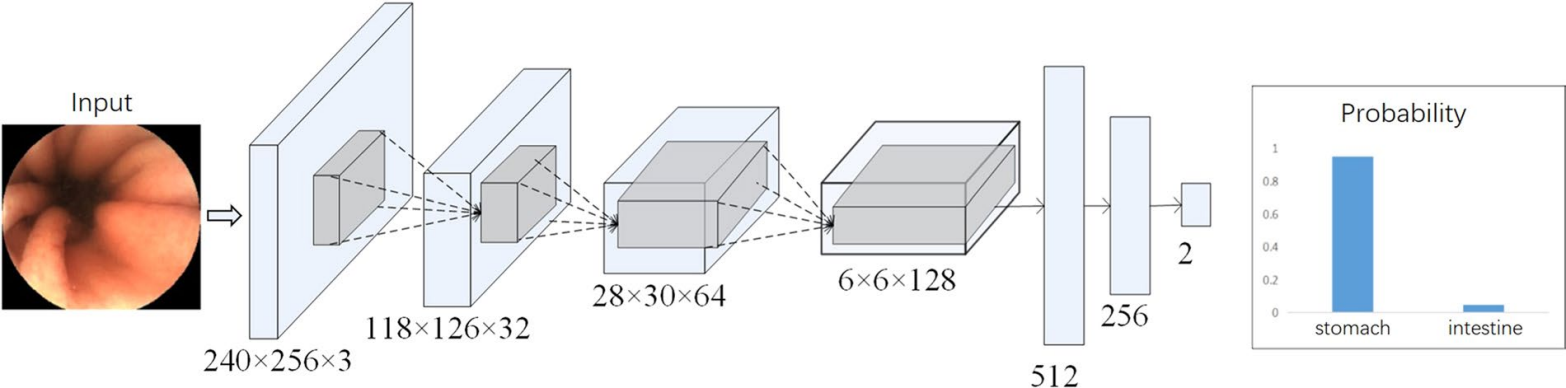
The neural network model. The endoscopic image classification network is a modified Convolutional Neural Network based on LeNet. The endoscopic image is sent directly to the network as an input, and the neural network can directly calculate the probability of the stomach and the small intestines. According to the probability score, it can be discriminated whether the input image is the stomach/duodenal bulb or the DSD.

To construct the AI-based diagnostic system, we utilized the deep neural network architecture modified from LeNet^24^, only altering the model configurations and not its algorithm. LeNet is the classical deep CNN model that consists of several convolutional layers and fully-connected layers. The configuration of the network (Fig. 4) can conduct binary classification tasks. There are some detailed configurations, i.e., kernel size = 5, stride = 1, pooling = max pooling, padding = “SAME”. The “ReLu” is used as the activation function, and the “focal loss” is chosen as the loss of the function for optimization. All the labels for the stomach and the duodenal bulb in the training set were annotated manually by the expert endoscopists. These images were put into the network through the TensorFlow implemented deep learning framework. The framework, developed from Google, was used to train and validate the CNN in this study. This framework is one of the most popularly- and widely-used frameworks. The CNN was taught to classify an image as either the stomach or the duodenal bulb.

All the layers of the CNN were trained by the Adam optimizer with a global learning rate of 0.0002, and the parameters with beta1 = 0.9 and beta2 = 0.999. The batch size was 64. The network was converged after 20 epochs. These hyper-parameters, e.g., learning rate, batch size, epoch number and parameters of optimizer, were set up through trial and error to ensure that all the data were compatible with our network.

Each image had the same resolution of 240 × 256 pixels. The original intensities of the images were between 0 and 255, which were normalized to [0, 1] by dividing with 255. Some data augmentations were also involved to improve the performance, including image mirroring that flipped the image horizontally and vertically. The image cropping and resizing could crop the image to 0.85 of width and height randomly and then resize back to the original resolution 240 × 256. The image contrast adjustment could multiply a value between [0.9, 1.1], and the brightness adjustment could add a global value ranging from [−0.05, 0.05] to a normalized image. The data augmentation was applied to each image randomly, where the image might receive none of the above data augmentation, one or several combinations of them.

Apart from the network structure (Fig. 4), we also tried several other network structures. Specifically, Fig. 4 contained 4 convolutional layers and 3 fully-connected layers (4-conv-3-fully). We also tried the networks consisting of 2-conv-2-fully, 3-conv-2-fully, 3-conv-3-fully, and 4-conv-4-fully. All these networks performed equally well when compared with ours (4-conv-3-fully). Specifically, the accuracy of these networks might drop 1–2 points in terms of classification accuracy, which had no influence on the final result. In particular, the networks of 2-conv-2-fully, 3-conv-2-fully, and 3-conv-3-fully were more shallow than ours, which might turn to a little bit under fitting while the network 4-conv-4-fully was deeper than ours, which could turn to a bit overfitting, causing the slight drop of the accuracy. In contrast, our network achieved the best performance. We also tried several other classic network structures, including VGG16^33^, VGG19^33^, ResNet18^34^, ResNet50^34^, all of which could turn to a bit overfitting, leading to the drop of the accuracy.

**Figure 5:**
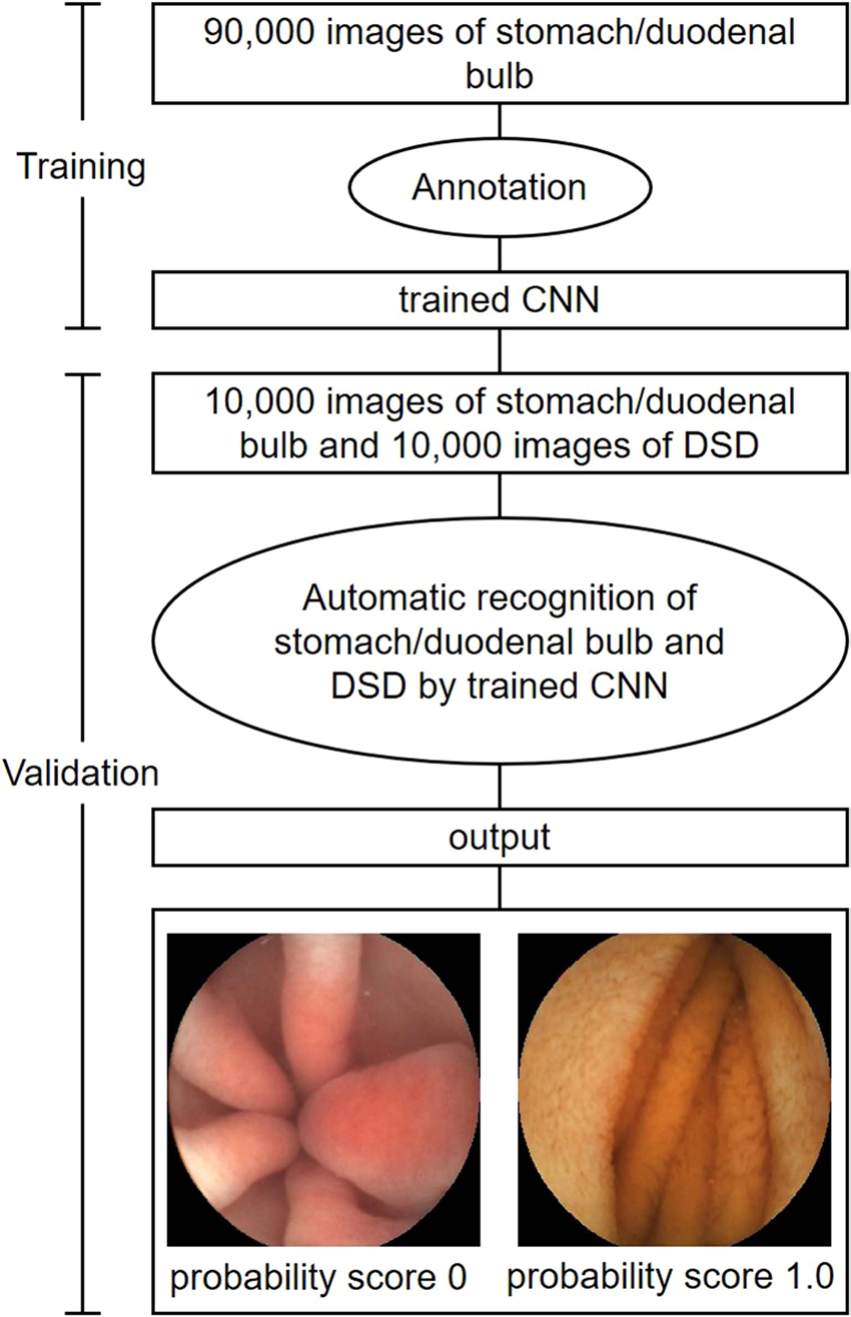
The flowchart of this study.

**Figure 6:**
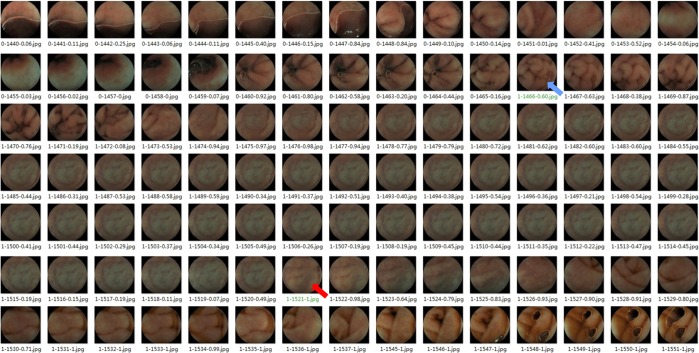
The illustration of the outcome judgement. The number of the filename of each image consisted of three parts, separated by two hyphens, of which the first part was the ground truth labels 0 or 1; 0 meant the stomach/duodenal bulb, and 1 meant the DSD, the second part was the consecutive image number, and the third part was the probability score, and it can be discriminated whether the image was the stomach/duodenal bulb or the DSD according to the cut-off value (0.42). The green filename pointed by the blue arrow was the outcome of the CE into the DSD judged by the endoscopists, and the outcome judged by the CNN pointed by the red arrow, and 54 images delayed (1521 Vs.1466), so the deviation time was 27 seconds delay (the filming speed of the CE was 500 ms/sheet).

**Figure 7:**
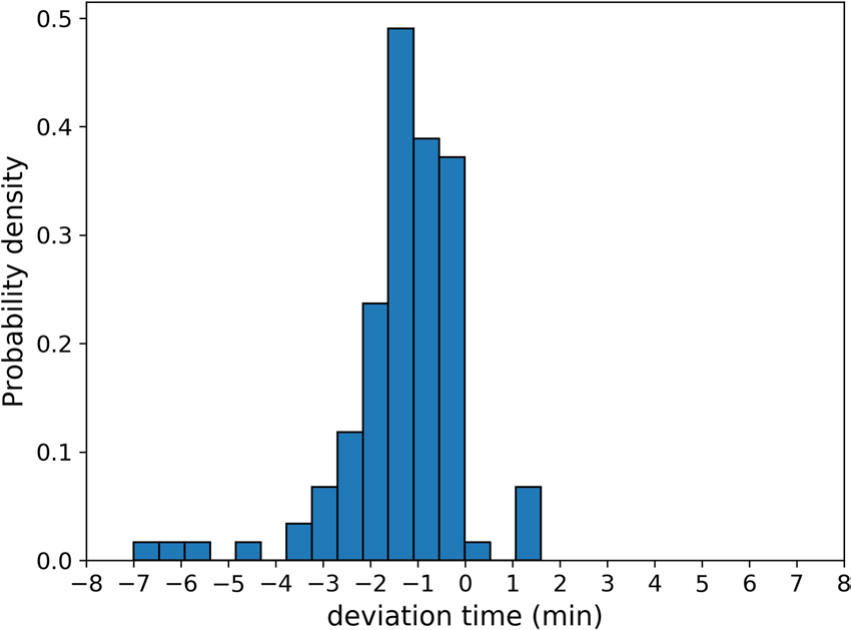
The illustration of the prediction error rate. The value in the X-axis was the deviation time (min). The negative value meant the deviation ahead of the time marked by the endoscopists, and the positive value meant the delay of the time marked by the endoscopists. The value in the Y-axis was the value of the probability density of the prediction error rate by the CNN in the different time interval.

In our study we used the computer with memory 32 G, Intel Core i7-6850K CPU @ 3.60 GHz, Solid State *Disk* 452GB, Nvidia GeForce GTX1080Ti, with a Ubuntu 14.04 Operation System. The flowchart of the study design was listed (Fig. 5) and the outcome judgement was listed (Figs. 6 and 7).
